# Findings from an exploration of a social network intervention to promote diet quality and health behaviours in older adults with COPD: a feasibility study

**DOI:** 10.1186/s40814-020-0553-z

**Published:** 2020-02-06

**Authors:** Ilse Bloom, Lindsay Welch, Ivaylo Vassilev, Anne Rogers, Karen Jameson, Cyrus Cooper, Sian Robinson, Janis Baird

**Affiliations:** 1 MRC Lifecourse Epidemiology Unit, Southampton General Hospital, University of Southampton, Southampton, SO16 6YD UK; 2 NIHR Southampton Biomedical Research Centre, University of Southampton and University Hospital Southampton NHS Foundation Trust, Southampton, UK; 3 Solent NHS Trust, Bitterne Health Centre, Commercial Road, Bitterne, Southampton, UK; 4 Faculty of Environmental and Life Sciences, School of Health Sciences, University of Southampton, Southampton, UK; 5 NIHR Collaboration for Leadership in Applied Health Research (CLAHRC) Wessex, Southampton, UK; 6 NIHR Musculoskeletal Biomedical Research Unit, University of Oxford, Oxford, UK; 7 AGE Research Group, Biomedical Research Building, Campus for Ageing and Vitality, Newcastle University, Newcastle upon Tyne, UK

**Keywords:** Ageing, Diet, COPD, Feasibility, GENIE, Health behaviours, Older adults, Randomised controlled trial, Social networks

## Abstract

**Background:**

Diet quality in older people with chronic obstructive pulmonary disease (COPD) is associated with better health and lung function. Social factors, such as social support, social networks and participation in activities, have been linked with diet quality in older age. A social network tool—GENIE (Generating Engagement in Network Involvement)—was implemented in a COPD community care context. The study aimed to assess the feasibility of the GENIE intervention to promote diet quality and other health behaviours in COPD.

**Methods:**

Twenty-two community-dwelling older adults with COPD were recruited from a local COPD service. Participants were offered usual care or the GENIE intervention. Process evaluation methods were used to assess intervention implementation, context and mechanisms of impact; these included observations of patient interactions with the intervention, documented in observational field notes and in films of a patient group discussion. Diet quality was assessed by food frequency questionnaire; ‘prudent’ diet scores were used to describe diet quality at baseline and at 3-month follow-up. Change in diet quality was expressed per month, from baseline to follow-up.

**Results:**

Feasibility data showed that the GENIE intervention could be implemented in this sample of community-living older people. The intervention was acceptable to clinicians and older people with COPD, especially for those with less severe disease, when facilitated appropriately and considering the levels of literacy of participants. There was no significant change in diet quality in the intervention group over the follow-up period (median change in prudent diet score per month (interquartile range (IQR), 0.03 (− 0.24–0.07)), whereas an overall fall in diet quality was observed in the control group (− 0.15 (− 0.24–0.03)).

**Conclusion:**

The process evaluation findings suggest that this intervention is feasible and acceptable to both patients and clinicians. Although the sample size achieved in this study was small, findings suggest that the intervention may have a protective effect against declines in diet quality, and other health behaviours, in an older COPD population. Findings from this feasibility study indicate that further evaluation of the GENIE intervention is warranted in a larger study, with a longer follow-up.

**Trial registration:**

ClinicalTrials.gov, NCT02935452. NIH U.S. National Library of Medicine. Registered 17 October 2016.

## Background

Maintaining adequate dietary intakes in individuals with chronic obstructive pulmonary disease (COPD), and indeed in older age, is key for health and wellbeing [1, 2]. Better diet quality, broadly indicating greater adherence to recommendations for a ‘healthy’ diet (for example, higher intakes of fruit, vegetables, oily fish and whole grains), has been associated with better lung health and lung function [1, 3], as well as reduction in disease risk and frailty [4, 5]. However, despite the recognised importance of diet for health in older age, there is evidence that poor diet quality is common in older adults, including in the UK [6–8]. Thus, there is a need for effective interventions to promote healthy eating among some older people living in the community.

Cross-sectional studies have found that older men and women who live alone have poorer diet quality than those living with a partner [8, 9]. Both living alone and having less frequent contact with friends exacerbated the effect of widowhood on decreasing vegetable variety, suggesting that support from friends may compensate for the lack of a partner [10, 11]. Social relationships could enhance resilience in older people, when these precede, and continue throughout, periods of adversity [12]. Moreover, involvement in leisure activities could become increasingly important with age and could contribute to resilience in older people [13]. Consistent with these messages, findings from a recent qualitative study suggested that social and psychological factors might mediate the influence of a range of background or contextual ageing-related factors (including bereavement, medical conditions and environmental factors, such as access to shops) on the diets of community-living older people [14]. This study posited that greater social engagement and stronger social relationships may offset the effects of some of the barriers to eating a healthy diet that often come with the ageing process.

The increasing evidence that social factors might be important influences on older people’s diets highlights the potential of aspects, such as social engagement, to be modifiable factors to include in strategies to enhance the diets of older people. Currently, there is limited consideration of social engagement in the design of interventions aimed at promoting healthy eating among older people [15]. Interventions to enhance diet quality in older age could add value to the long-term health of older people and those with long-term conditions in the community. Indeed, improving health behaviours, including diet, in patients with COPD could be a valuable clinical intervention for managing the condition.

In the present study, the GENIE (Generating Engagement in Network Involvement) social network intervention tool was used in a randomised controlled trial feasibility study. GENIE is designed to work by ‘initiating positive disruption of established self-management practice through mapping of and reflection on personal network membership and support’, which presents ‘possibilities for reconstructing self-management differently from current practice’ [16]. The GENIE intervention has been shown to improve engagement with resources and connections that support self-management in people with type 2 diabetes [16]; furthermore, there are indications of a positive impact on health outcomes and quality of life [17].

The Medical Research Council framework for complex interventions sets out various phases in the process of development and evaluation, all the way through to post-evaluation implementation, of a complex intervention [18]. One of the key phases of this process is assessing feasibility of the intervention, so as to test procedures, determine acceptability of the intervention and estimate recruitment and retention. In keeping with this framework, we carried out a feasibility study and an exploratory evaluation of the intervention, in the context of an older population with debilitative respiratory disease, which can restrict mobility and confidence due to tissue wasting and episodes of breathlessness; the next phase would be to conduct a definitive evaluation of the intervention.

Using a randomised controlled trial (RCT) design, the aims of this feasibility study were (1) to pilot outcome data collection methods and develop process evaluation methods that could be used in a larger study, (2) to assess the feasibility of scaling this study up into a larger future study and (3) to assess the potential impact of GENIE on diet quality, and other health behaviours and health outcomes, in a group of older community-dwelling adults with COPD and to compare changes in the outcomes of interest with those in a control group. This feasibility study intended to clarify various aspects, including the number of eligible patients; the willingness of clinicians to recruit participants and the willingness of participants to be recruited and randomised (recruitment); follow-up rates, response rates to questionnaires (retention); the practicality of delivering the intervention in a COPD clinic; and the acceptability of the intervention to older adults with COPD, to understand what adaptations might be required in this population. In this feasibility study, we carried out a small randomised controlled trial for the main purpose of testing trial processes, and also the potential of the intervention.

## Methods

Patients were recruited from the east of Southampton, which covers the areas with some of the highest deprivation in Southampton city [19] and COPD prevalence [20]. Patients were selected for recruitment during attendance at the local COPD pulmonary rehabilitation programme. Information about the study was provided in either an accessible information format or the usual format for patient information.

Patients aged from 18 to 95 years of age, with a diagnosis of COPD, living in the east of Southampton were eligible to participate. Patients of all COPD disease severities were included. Patients without a clear COPD diagnosis or unable to give informed consent were excluded. Patients lacking fluent English language, on an end of life pathway, or with major psychological illness were also excluded.

Community patients were booked for a baseline visit at a local health centre, where informed consent was obtained, baseline questionnaires were administered and participants were randomised. Participants in the control group received usual clinical care (discharge planning with suggested activities, usually exercise therapy), and those in the intervention group received the GENIE intervention (in addition to usual clinical care). Block randomisation was used, a commonly used technique in clinical trial design that aims to reduce bias and achieve balance in the allocation of participants to treatment arms [21]. To further reduce unconscious bias of the researcher, pre-prepared envelopes containing the possible combinations of group allocation (group A, intervention, or B, control) were stored in a locked drawer on site at the health centre.

**Fig. 1:**
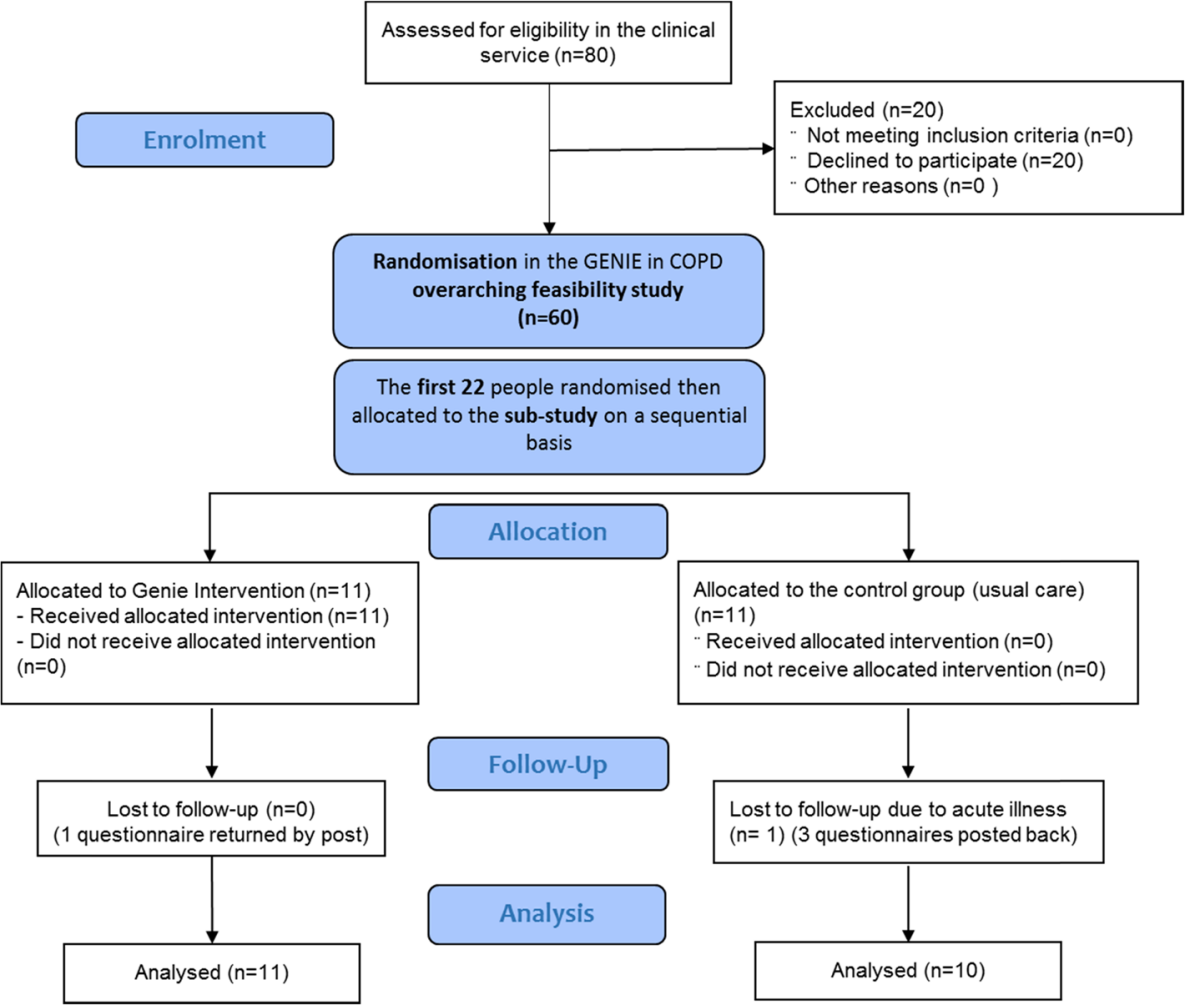
CONSORT flow diagram for the feasibility study [23]

This feasibility study was a sub-study embedded in a larger feasibility study that aimed to implement and evaluate the use of the GENIE intervention tool in a Southampton Integrated COPD Service, to ascertain potential cost-effectiveness and patient benefit. The main outcomes of this larger study were social network diversification, healthcare utilisation, burden of disease, psychological outcomes and quality of life. In addition to the wider aim of assessing the feasibility of the GENIE intervention in an older population with COPD and of scaling this study up into a larger future study, the aims of the present sub-study included trying out an additional questionnaire to collect data on health behaviours (only these outcome data were analysed here) and developing process evaluation methods that could be used in a full-scale study, consistent with MRC guidance on process evaluation of complex interventions [22]. In this feasibility study, process evaluation plays an important role to understand the practicability of the intervention and to make any necessary adaptations to its design and evaluation in a future full-scale study to evaluate the impact of GENIE on diet and other health behaviours. Participation in the present feasibility sub-study was optional, as it included a further questionnaire that may have added a burden to some participants. In order to prevent biased selection, participation was offered on a first-come-first-serve basis; in this way, the sub-group was also part of the randomisation process above. The sub-study aimed to recruit around 30 people to understand feasibility of the study and usability of the questionnaires. However, due to the large amount of detailed data collection required for the sub-study, after 22 patients had been interviewed, and in discussion with other members of the study team, it was deemed that sufficient data had been gathered. Figure 1 shows the CONSORT flow diagram, which also depicts the relationship between this study and the larger study. The CONSORT 2010 feasibility study checklist has been appended (see Additional file 1).

A follow-up visit was booked approximately 3 months (12 weeks) from the day of the baseline visit. Participants were invited back, via letter and/or phone call, to attend the 3-month follow-up visit at the local health centre. At this visit, questionnaires were administered to collect follow-up data.

### The Generating Engagement in Network Involvement (GENIE) intervention

Kennedy and colleagues developed the GENIE intervention using an evidence-based and theoretically driven approach [16], moving away from the more individualised models of self-management support and behaviour change, towards a more collectively orientated approach, with recognition of the social and environmental influences on self-management and health behaviours. The social network approach has been shown to improve health-related outcomes [16]. The GENIE intervention and web-based tool were developed to take a multi-level, network approach to self-management support, to ‘improve people’s ability to navigate and negotiate support available from within personal social networks and extend this to engagement with local groups and organisations’ [16, 24].

**Fig. 2:**
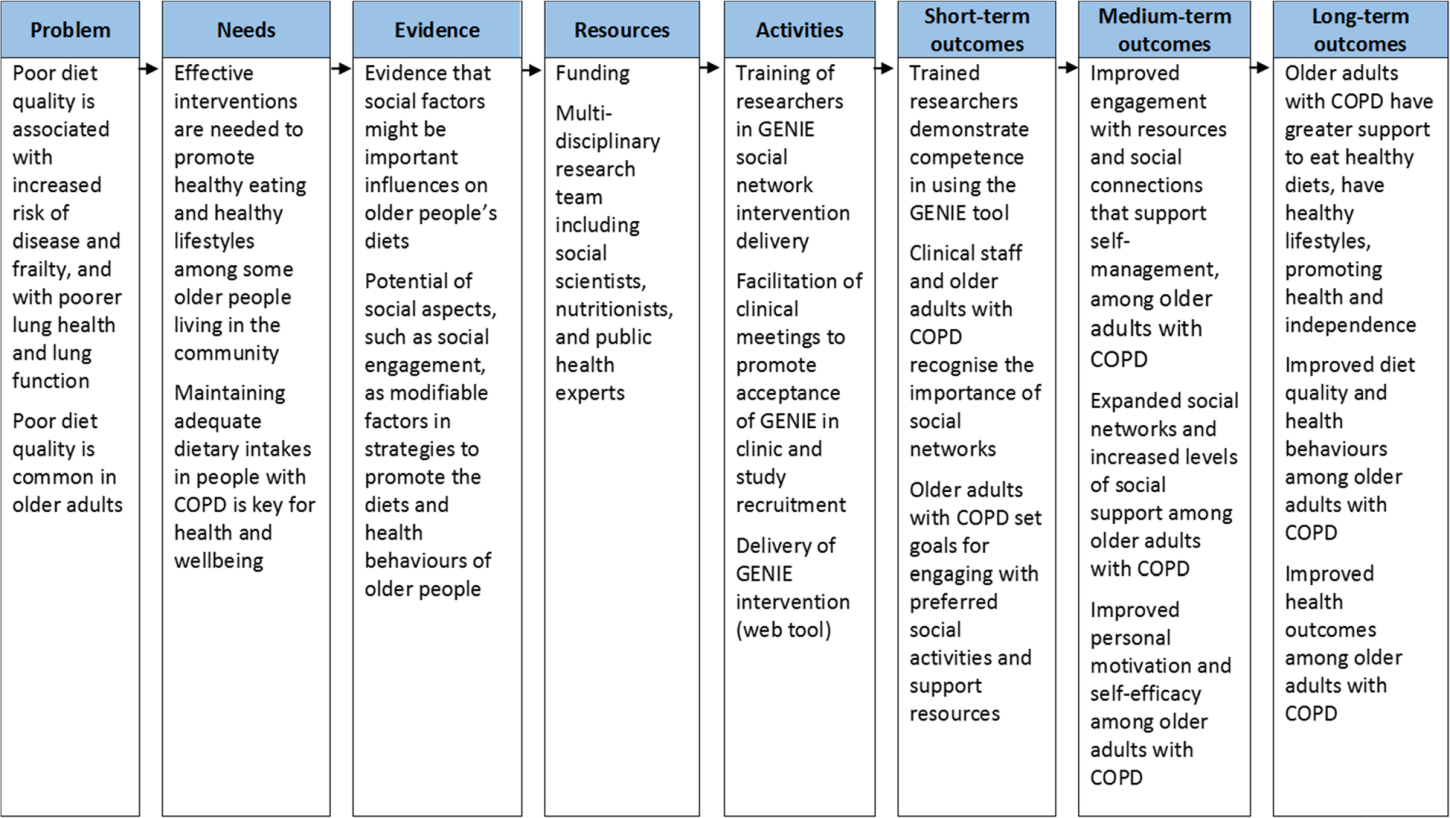
Logic model for the GENIE intervention, to promote diet quality and health behaviours in community-living older adults with COPD

The GENIE social networking tool is a facilitated online tool, designed to map an individual’s network of support, for reflection on its composition, to elicit preferences and signpost the individual to valued social activities. The tool has a database, which, for the purposes of the broader study in which this feasibility study was nested, was manually programed with COPD-specific (plus existing generic) online and offline resources, groups and organisations. The organisations were recognised charitable and clinical support groups local to the east of Southampton and local community groups recommended by the Itchen Region Councillor, who was supportive of the development of community solutions for local residents. Figure 2 shows the GENIE intervention in a logic model to elucidate the theoretical underpinnings, in terms of promoting diet quality and health behaviours in community-living older adults with COPD.

The process of delivering the GENIE intervention can be broken down into distinct stages [16] (for visual representation of the stages and examples, see Additional file 2):

- Stage 1: The participant is supported through a mapping process of their current social support with the facilitator, using a concentric circles approach.
- Stage 2: The concentric mapping promotes conversation to elicit values and key preference questions in the intervention highlight preferred activities and support resources.
- Stage 3: Linking individuals to prioritised and valued activities and resources (links are to a pre-created database where local organisations and resources have been categorised).
- Stage 4: The GENIE tool then presents options in a user-friendly way, on a Google map with clear details about access.

The GENIE tool was delivered face-to-face using a computer, by trained researchers (LW and CA). The delivery of the GENIE intervention took 45 min to 1 h. Participants had the option to have a link sent to their email to log into the website at a later date if they wished.

Usual care was also provided to the GENIE intervention group and the control group. Usual care consisted of the pulmonary rehabilitation discharge pack, containing a British Lung Foundation (BLF) exercise DVD and guidance, information about local BLF Breathe Easy support groups and local walking groups.

### Outcome measures

Quantitative outcome measures were collected at baseline and at the 3-month post-intervention follow-up visit.

Diet was assessed using a short food frequency questionnaire (FFQ), which has been developed to assess diet quality in older adults [25]. In this feasibility study, ‘prudent’ diet scores were calculated for each participant at baseline and follow-up, based on their consumption of 19 foods, that indicated the participant’s compliance with the ‘prudent’ dietary pattern, and was used as an indicator of diet quality [25]. High prudent diet scores indicate diets characterised by frequent consumption of fruit, vegetables, wholegrain cereals and oily fish but low consumption of white bread, added sugar, full-fat dairy products, chips and processed meat [25]. Changes in prudent diet scores (representing change in diet quality) were expressed per month, from baseline to follow-up.

Further outcome measures included participant-reported changes in alcohol consumption, smoking status, body mass index (BMI), appetite, physical function and total physical activity. Expressed per month, from baseline to follow-up, change in alcohol consumption and smoking was expressed over the entire follow-up period.

Height (cm) and weight (kg) were obtained from participants’ most recent clinical records, usually within the preceding 6–9 months, or weighed at baseline and BMI (kg/m^2^) was calculated for each participant. Appetite was assessed using the Simplified Nutritional Appetite Questionnaire (SNAQ), which has been shown to predict weight loss in community-dwelling older people [26]. Data on physical function were collected using self-reported assessment of physical function (SF-36 physical functioning (PF) domain—SF-36 PF); poor physical function was defined as being in the bottom sex-specific fifth. Data on physical activity were collected using the International Physical Activity Questionnaire (IPAQ) Short Form; in accordance with IPAQ, physical activity level was calculated and categorised as either low, moderate or high activity [27].

At baseline, demographic data were also collected from participants on age, gender, age they left school, highest level of qualification attained, job or occupation, the number of people living in the participant’s household and the number of regular visitors received. FEV_1_ values were recorded to ascertain the level of COPD severity.

### Statistical analysis

Baseline and follow-up descriptive characteristics were reported as mean with standard deviation (SD) for continuous normally distributed variables, median with interquartile range (IQR) for continuous variables with a skewed distribution or counts and percentages for categorical variables, as appropriate. Descriptive statistics for health behaviours and health characteristics were presented separately by participant group (intervention and control). Given the small sample size of this feasibility study, there was a lack of statistical power to detect differences between the participants groups (intervention vs. control); statistical tests would be performed in an adequately powered future full-scale study. Data were analysed using Stata version 14.2.

### Process evaluation

The process evaluation of this study is guided by the Medical Research Council guidance on process evaluation of complex interventions [22]. At the feasibility stage, process evaluation is essential to understand the viability of the intervention and to optimise its design and evaluation for a full-scale study [22]. We assessed the implementation of the GENIE intervention (in terms of reach, recruitment and retention, fidelity, dose offered, adaptations and dose received), the mechanisms of its impact and context, in interviews with participants and clinicians in the COPD service. Observations of participant interactions with the intervention, during the introduction of the intervention to participants, and the implementation and delivery of the intervention, were captured by one of the researchers (LW). These were documented as observational field notes and captured in video recordings (see Additional file 3) during informal discussions with a group of participants who had received the GENIE intervention and were part of a COPD support group. Further observations were made at clinical multi-disciplinary team meetings, in which the GENIE intervention and online tool were introduced to clinicians. Field notes of subsequent conversations with clinicians were also collated. These observations were discussed with and triangulated by a second researcher (AR), who also attended clinical team meetings.

Field notes were written into an ethnographical interpretivist account by LW. This account was shared with another researcher (IB) and reviewed in conjunction with the video recordings in order to evaluate the implementation process and draw conclusions regarding the acceptability of the intervention. In terms of the contextual component of the process evaluation, we aimed to assess the context into which the intervention was introduced. The field notes and observations that were collected spanned the whole process, ranging from early introduction and acceptability of the GENIE tool to the clinical team, to the political and cultural context of the healthcare setting and the process of delivery. In order to enable broader implementation of the GENIE tool in the COPD service (the aim of the wider study), change and negotiations were required on multiple levels of the organisation. Local groups and resources in the community around the COPD service in which the patients lived and worked were assessed by a researcher (LW) and, if appropriate, were added into the GENIE resource database. To facilitate an understanding of the mechanisms of impact of this intervention, participant uptake of social activities was recorded, using the GENIE tool, for comparison between baseline and follow-up. For those in the intervention arm, feedback and reflection discussions were initiated using the network diagrams.

## Results

Twenty-two people were recruited to this study; 11 participants were randomised to each group, and 1 participant was lost to follow-up in the control arm (Fig. 1 shows the study CONSORT diagram). For a small number of participants in the control group, there are missing data at follow-up, due to participant time commitments outside the study and reported questionnaire fatigue. Some participants were unable to attend a follow-up appointment and therefore questionnaires were posted to them; in some cases, not all the pages of the questionnaires were completed and some were missed. However, all data relating to diet was captured.

Tables 1 and 2 show the baseline characteristics of the study population. Table 2 shows baseline and follow-up characteristics of study participants by group, and there is a description of baseline health behaviours and other characteristics, for the whole study population combined, in the text below. At baseline, participants were aged between 61 and 82 years and 41% of participants lived alone. In terms of their COPD disease severity, for half of participants, this was moderate (*n* = 11), while for 36% (*n* = 8), it was severe. While all participants had smoked at some stage during their lives, only 9% (*n* = 2) smoked at the time of baseline data collection. At baseline, most study participants (82%) consumed alcohol, median BMI was 25.7 kg/m^2^ (IQR 21.7–29.5), and over a third (36%) of participants had poor appetite. Over a quarter (27%) of participants had low physical activity, at baseline. In this small group, given the sample size, there was a lack of statistical power to detect differences between intervention and control groups at baseline.

**Table 1:** Baseline characteristics of the study cohort—background characteristics

	All			Intervention			Control		
	*N*	Median	IQR	*N*	Median	IQR	*N*	Median	IQR
Age	22	70	67–77	11	70	68–71	11	77	66–82
	Total *N*	*N*	%	Total *N*	*N*	%	Total *N*	*N*	%
Gender	22			11			11		
Male		13	59.1		8	72.7		5	45.5
Female		9	40.9		3	27.3		6	54.6
Age left school—category	22			11			11		
< 15		3	13.6		0	0		3	27.3
≥ 15		19	86.4		11	100		8	72.7
Highest qualification—category	21			10			11		
None		4	19.1		1	10		3	27.3
O/A levels/vocational qualifications		14	66.7		6	60		8	72.7
Degree or higher		3	14.3		3	30		0	0
Job/occupation—category	21			10			11		
Manual		11	52.4		5	50		6	54.6
Non-manual		10	47.6		5	50		5	45.5
Number of people in household	22			11			11		
1		9	40.9		4	36.4		5	45.5
2		13	59.1		7	63.6		6	54.6
	*N*	Median	IQR	*N*	Median	IQR	*N*	Median	IQR
Regular visitors	22	4	2–6	11	4	3–6	11	4	2–7
	Total *N*	*N*	%	Total *N*	*N*	%	Total *N*	*N*	%
Disease severity^a^	22			11			11		
Mild		2	9.1		2	18.2		0	0
Moderate		11	50		6	54.6		5	45.5
Severe		8	36.4		3	27.3		5	45.5
Very severe		1	4.6		0	0		1	9.1

^a^Disease severity (categorised as mild, moderate, severe or very severe based on the GOLD classification [28])

**Table 2:** Health behaviours and other health characteristics of participants, by group, at baseline and at follow-up

	Intervention group						Control group					
	Baseline			Follow-up			Baseline			Follow-up		
	Total *N*	*N*	%	Total *N*	*N*	%	Total *N*	*N*	%	Total *N*	*N*	%
Currently drink alcohol	11	8	72.7	11	7	63.6	11	10	90.9	8*	6	75.0
Currently smoke	11	1	9.1	11	1	9.1	11	1	9.1	8*	1	12.5
	*N*	Median	IQR	*N*	Median	IQR	*N*	Median	IQR	*N*	Median	IQR
BMI (kg/m^2^)	11	26.5	21.7–29.5	11	26.3	21.9–29.4	11	24.2	20.7–30.1	8	23.0	21.3–29.7
Prudent diet score	11	0.31	−0.61–1.26	11	0.47	−0.04 – 0.70	11	0.96	0.26–1.71	10**	0.39	−0.16 – 0.97
Total SNAQ score	11	16	13–18	11	14	11–16	11	14	12–16	7*	13	11–16
	Total *N*	*N*	%	Total *N*	*N*	%	Total *N*	*N*	%	Total *N*	*N*	%
SNAQ category^a^	11			11			11			7*		
SNAQ score < 14		3	27.3		4	36.4		5	45.5		4	57.1
SNAQ score ≥ 14		8	72.7		7	63.6		6	54.6		3	42.9
Physical activity category^b^	11			11			11			10**		
Low activity		3	27.3		5	45.5		3	27.3		4	40.0
Moderate activity		4	36.4		2	18.2		3	27.3		3	30.0
High activity		4	36.4		4	36.4		5	45.5		3	30.0
	*N*	Median	IQR	*N*	Median	IQR	*N*	Median	IQR	*N*	Median	IQR
Total physical activity^c^	11	520	240–1500	11	280	60–1680	11	920	210–1560	9*	245	150–440
Physical function score (SF-36)	11	30	15–55	10*	40	15–55	11	25	20–50	8*	30	15–40
	Total *N*	*N*	%	Total *N*	*N*	%	Total *N*	*N*	%	Total *N*	*N*	%
Poor physical function^d^	11	3	27.3	10*	3	30.0	11	2	18.2	8*	2	25.0

^a^Total SNAQ (Simplified Nutritional Appetite Questionnaire) score < 14 (low appetite)

^b^Physical activity scores were categorised into three categories (low, moderate or high activity)

^c^Total physical activity performed, in minutes, per week

^d^Physical function scores (SF-36) were categorised to reflect whether or not participants had ‘poor physical function’ (if their physical function score was in the sex-specific bottom fifth of the distribution)

*Missing data due to questionnaire non-completion

**One participant in the control group dropped out of the study due to hospitalisation

### Outcome analysis

Median change in prudent diet score (per month) in the whole cohort was − 0.09 (IQR − 0.24–0.06). There was no significant change in diet quality in the intervention group over the period of follow-up (median change in prudent diet score per month inter quartile range (IQR) 0.03 (− 0.24–0.07)), whereas an overall fall in diet quality was observed in the control group (median change in prudent diet score per month (IQR) − 0.15 (− 0.24–0.03)). Although this is suggestive of beneficial effects of the intervention on diet quality, the sample size was limited to detect differences between the groups (Table 3).

**Table 3:** Assessment of the change in outcome variables, between baseline and follow-up, in the intervention and control groups

Outcome^1^	Intervention group	Control group
Change in prudent diet score	0.03 (− 0.24 to 0.07)	− 0.15 (− 0.24 to 0.03)
Change in BMI	− 0.02 (− 0.42 to 0.24)	0.18 (− 0.13 to 0.20)
Change in appetite score^a^	− 0.23 (− 0.89 to 0)	0 (0 to 0.45)
Change in physical function score^b^	0 (− 2.16 to 3.71)	−2.46 (− 3.38 to 0.55)
Change in total physical activity^c^	− 51.81 (− 66.81 to 54.91)	−167 (− 365.62 to − 29.53)

^1^All change outcomes are expressed per month, from baseline to follow-up, as median (IQR)

^a^Change in total SNAQ (Simplified Nutritional Appetite Questionnaire) score

^b^Change in physical function score (SF-36)

^c^Change in total physical activity performed, in minutes, per week

There were few current smokers and there was little change in smoking or alcohol consumption status.

There was little change in BMI in either the intervention or control groups over the follow-up period. In the intervention group, there was an overall small decline in appetite over the follow-up period, whereas in the control group, there was no change in appetite (Table 3). There was no change in physical function in the intervention group over the period of follow-up, while an overall decline in physical function was observed in the control group (Table 3). Over the follow-up period, there was an overall fall in total physical activity performed by participants, in both the intervention and the control groups; however, the fall was most pronounced in the control group.

### Findings from the process evaluation

The implementation of the GENIE intervention was assessed during this feasibility study. Participants were willing to be recruited into the study and appeared to accept the concept of a social tool and recognise the value of social interactions in their disease management. Participants appeared uplifted by the options of choice offered by GENIE and the recognition of the importance of their social world; participants enjoyed discussing their social world rather than constantly focussing on their condition. Participants in the intervention group with less severe disease (mild or moderate) (*n* = 8, 72.8%) were pleased that they had been given permission to socialise more. Participants indicated that the delivery of the intervention suggested to them that clinicians recognised the value of personal social interactions beyond illness management.

However, in the intervention group, those with severe disease (*n* = 3, 27.3%) or experiencing frequent exacerbations reported that the intervention was hard to engage with, as their main goal was to ‘feel better’. As observed and reported in the field notes, discussions with study participants showed that some of them had low levels of literacy and the language used by GENIE was difficult for many to understand without facilitated support. This points to the key role of the facilitator and the face-to-face delivery of the GENIE intervention, which is in line with previous research [16]. Researchers had to read aloud for many of the GENIE tool’s online aspects, which made intervention delivery more difficult and time-consuming, as such, in terms of intervention fidelity, the intervention delivery needed to be adapted and the dose of the intervention required adjustment in this population. Some of the participants found it tiring to complete the baseline questionnaires, as well as the online GENIE tool. Researchers found that using lay language to explain the intervention approach (e.g. using the expression ‘circle of friends’) proved more successful than using the conventional wording on the participant information sheet (PIS). Therefore, an accessible information sheet was prepared and approved to facilitate participant recruitment and retention. The language used in the conventional PIS referred to the study as a student research project; this was reviewed by patients in the service during the recruitment process, who commented that this indicated to them that the study was being conducted for personal gain, rather than patient benefit, and the term ‘student’ appeared to them to confer less credibility to the study. Furthermore, the digital literacy of participants was also poor; most requested that everything be printed on paper and declined to have the option to log on and use GENIE for themselves online and to interact with any form of technology, and this was another adaptation of the intervention delivery in this population, which likely affected the dose of the intervention received. However, the facilitation of GENIE, by talking through the process using lay language and providing paper-based printouts of their chosen activities, overcame this for the majority of participants. From our observations, the facilitation process appeared to be cathartic for many of them.

In a discussion with participants, they indicated what they valued about their interactions with GENIE.

Often people are told they’ve got COPD they go home just sit in the chair and do nothing, therefore the illness takes over, and you just become worse and worse, just wallow in your own self-pity … Video quote 1

The GENIE process also encouraged participants to take a step further into friendships, with positive reciprocal gains.

I got all these forms of all different places that I can go in the area which are free … walking and knitting … the GENIE, the idea is for people on their own that don’t go out and don’t go nowhere, and meet up with people … it’s a social circle, the bullseye of the social circle gets bigger … Video quote 2

If you’re not feeling very well who do you turn to? My mates. … Well family and that are all working … Video quote 3

In terms of the context into which the intervention was introduced, clinicians were introduced to the tool prior to the study start. They were initially sceptical, as the tool was patient-led, with the patients guiding the choices of socialisation options, rather than being clinically directed. Over time, and with key clinicians championing the tool, the clinical team started directly referring or signposting patients to the study as they recognised the need for a social intervention as part of COPD patients’ usual care. It was through this engagement with the intervention that they were able to reflect on the value of patient networks and the impact that personal social circumstances can have on long-term health, enabling a more holistic clinical appraisal of the multiple needs of patients during routine clinical consultations. Clinician engagement with the GENIE intervention enabled them to develop a more nuanced understanding of its value (e.g. for who it can work and under what circumstances). Below are quotes from two different COPD clinicians, who spoke to the researchers delivering the intervention at the time of participant recruitment. The first quote is from a clinician who is reflecting on their first consultation addressing the social elements of personal care, where they recognised the need for the GENIE social intervention at an earlier stage. The second quote is from a registrar who started to recognise the value of GENIE as a social network intervention.

I have just seen a person who is beyond GENIE. It is so desperately sad that his social world is so confined. He only sees one person, he has no friends and his ex-wife recently died. He feels he no longer has a reason to live. He used to feel comforted to know that his ex-wife was there and alive, even though they didn’t interact. I think GENIE is too much, how can we support this man socially? Clinician Quote 1

I have suggested GENIE for this person. There have social needs, and are isolated. I have documented this in their notes. Could you see them please? Clinician Quote 2

The final component of the process evaluation was an assessment of the mechanisms of impact of the intervention. Analysis of the data that was recorded on participant uptake of social activities, to assess the extent to which the intervention might have led to greater engagement with community resources and activities, was not completed for inclusion in this article. However, from discussions with some participants, it seemed that mapping their social world and talking through the concentric circle diagrams were seen as a positive disruption, potentially enough to initiate change in existing habits of socialisation and breaking routines that encroach in long-term conditions.

## Discussion

This paper describes a feasibility study of the GENIE social networking tool used in a population of patients with COPD. The study piloted outcome data collection methods and contributed to the development of the process evaluation methods, both of which could be used in a definitive intervention study. The study assessed feasibility of the GENIE tool in terms of clinician and patient acceptability and the feasibility of up-scaling into a larger future study. Overall, the study was received positively by participants. Clinicians required time and evidence to fully accept the concept of socially supportive methods into their routine clinical practice. Observations and discussions with clinicians and participants showed that there was a need to address literacy of the study participants and to simplify or modify the language used to introduce GENIE to make it easier to understand in this context.

Process evaluation findings indicate that the health literacy and other characteristics of participants should be an important consideration in the design of a future study. The assessment of intervention implementation suggested that severity of disease may impact the level of engagement with the intervention, including the ability to participate in social/community activities. It is possible that study participants with severe disease interpreted the severity of their COPD symptoms as a crisis; withdrawal from social networks and reduced network engagement can occur in a time of crisis, as a form of self-preservation, and avoidance of difficult relational work [29]. Hence, there may be a need for an adapted version of the GENIE intervention where the emphasis for people with a higher need for clinical support is not on expanding networks, but rather on reflection on current level of engagement and on the retention of existing social ties. For all participants, thinking or talking through the GENIE mapping tool enabled them to visualise their network and reflect on connections and understand where there might be gaps in social support. A further finding was that early engagement with clinicians, in the conceptual phase, provided a time frame for discussion and reflection on the study design and conceptualisation of a social network approach to the promotion of health behaviours. Overall, engagement with GENIE was found to be useful for clinicians in the sense that it offered a tangible and manageable process that they could engage with and reflect on the social context of patients. The GENIE tool process evaluation provided valuable insights into the context, reach and accessibility of the tool.

Using a randomised controlled trial design, the study also assessed the impact of GENIE on diet quality, and other health behaviours and health factors, in a group of community-dwelling older adults with COPD and compared changes with those in a control group. Although the sample size achieved was relatively small, the findings suggested potential protective effects of the intervention on diet quality, physical activity and physical function. While in the intervention group there was no change in diet quality over the period of follow-up, an overall decline in diet quality was observed in the control group. For physical function, there was no change in the intervention group over the period of follow-up, while an overall decline was observed in the control group. In addition, while in both groups there was an overall fall in total physical activity performed by participants, the fall was most pronounced in the control group. Against a background of worsening health behaviours, the intervention may have had a protective effect against declines in diet, physical function and physical activity in this population.

While there is some evidence to suggest that social involvement (e.g. links to community groups or organisations) may be associated with the maintenance of healthy behaviours over time in older people [30], there have been few intervention studies with a focus on social components and community engagement that have assessed impact on health behaviours, including diet, in older age.

In the present study, it is not clear why diet quality declined among control participants during the study or why changes occurred in some of the secondary outcomes (including physical function and physical activity) over the course of the study. The study was underpowered to detect differences that might exist between participants in the intervention group and those in the control group, at baseline. Despite random allocation to intervention and control groups, there did appear to be some baseline differences between them. Participants in the control group appeared older than those in the intervention group (median age 77 vs. 70 years), and they had a lower level of education (27.3% vs. 0% left school < 15 years; 27.3% vs. 10% had no qualification; 0% vs. 30% had a degree or higher qualification). At baseline, participants in the control group appeared more likely than those in the intervention group to live alone (45.5% vs. 36.4%) and were also more likely to have poor appetite (45.5% vs. 27.3%). Furthermore, participants in the control group appeared more likely than those in the intervention group to have severe or very severe disease at baseline (54.6% vs. 27.3%). It is possible that these differences could potentially account for the decline in diet quality, physical function, and the greater decline in physical activity that were observed among control participants during the study, compared to the maintenance of diet quality and physical function, and overall smaller decline in physical activity, in intervention group participants.

### Strengths and limitations

The observational data, patient videos and field notes used in the process evaluation provided insight into the feasibility of the intervention, including the clinical and patient acceptability of the implementation of this novel tool in a clinical setting. The process evaluation helped to identify barriers and challenges of implementation and possible adaptations that could enhance the design in a full-scale trial (e.g. accessible information, choice of language, possible clinical co-production). The measures that were used to assess the quantitative outcomes were based on self-reported data (except for BMI, for which height and weight were obtained from participants’ clinical records or participants were weighed). However, despite their self-reported nature, the measures used to assess diet quality, appetite, physical activity and physical function have been shown to be valid measures within older populations [25–27, 31]. The overarching RCT design is a strength of this study, with the presence of the comparison group helping to clarify what the intervention effects were.

However, researchers collecting the baseline and follow-up data also delivered the intervention, so they could not be blinded to the intervention status of participants. The sample size in this feasibility study was small, so it was less likely to detect differences that might exist between intervention and control groups. For a small number of participants in the control group, there were missing data at follow-up, but there were complete data for diet quality, the main outcome of this study. It is also possible that the follow-up period of 3 months was too short to capture significant changes in diet and other health behaviours; further data collection, in a larger sample with a longer follow-up period, would help to explore longer term behavioural changes. At this feasibility stage, mainly qualitative methods were used for the process evaluation. The methods could be expanded upon for the process evaluation of a larger intervention study. In addition to implementer self-report, semi-structured qualitative interviews could be conducted with participants and clinicians to assess implementation, context and mechanisms of impact. Qualitative measures that include structured observations and audio recordings of the intervention delivery could also be used. In a full-scale study, in addition to recording participant uptake of social activities using the GENIE tool (for comparison between baseline and follow-up), it would be important to collect quantitative data on potential mediating social and psychological factors (see Fig. 2) (e.g. measures of social networks, participation in social activities, social support, self-efficacy and motivation), to test hypothesised pathways.

## Conclusions

The process evaluation findings of this study suggest that it is feasible and that the intervention is acceptable to both patients and clinicians. Implemented in a local COPD service, the GENIE intervention was found to be acceptable and appropriate for older people with COPD, especially for those with less severe disease, when delivered by trained researchers. Overall, this feasibility study suggests that the GENIE tool can help people to think about the links they have with others (local groups, friends, family members, professionals) and to reflect on their involvement in social activities.

Although the sample size achieved in this study was small, the findings suggest the potential for protective effects of the GENIE intervention on diet quality, physical function and physical activity. However, it is not clear why diet quality, physical function and physical activity declined among control participants during the study. The 3-month follow-up period of this study was likely too short, and further evaluation is needed in a larger, more diverse group of community-dwelling older adults, with a longer follow-up period, to evaluate how social network interventions could be used to improve diet and health behaviours in older adults with COPD, therefore preventing declines in nutritional status and associated health consequences.

## Supplementary information

**Additional file 1.** CONSORT 2010 checklist.

**Additional file 2: Figure S1.** The stages of the GENIE intervention delivery. **Figure S2.** An example of a concentric circles diagram, which is used to facilitate the mapping of a user’s current support networks (Stages 1 and 2). **Figure S3.** An example of suggestions of local resources that are presented to GENIE users, with location and further details (Stage 4)

**Additional file 3.** Video clips used for observational data

## Data Availability

The study data from the over-arching clinical trial can be accessed via the ClinicalTrials.gov site as above and via the Dryad data repository.

## Abbreviations

BLF: British Lung Foundation
BMI: Body mass index
COPD: Chronic obstructive pulmonary disease
FEV_1_: Forced expiratory volume in one second
GENIE: Generating Engagement in Network Involvement
IQR: Interquartile range
NHS: National Health Service
NIHR: National Institute for Health Research
SNAQ: Simplified Nutritional Appetite Questionnaire
VC: Forced vital capacity
