# Supplementary material for: Findings from an exploration of a social network intervention to promote diet quality and health behaviours in older adults with COPD: a feasibility study
Source: Pilot Feasibility Stud. 2020 Feb 6;6:15. doi: 10.1186/s40814-020-0553-z (PMC7003327; doi:10.1186/s40814-020-0553-z)
Supplement: Supplementary file 2 — Additional file 2: Figure S1. The stages of the GENIE intervention delivery. Figure S2. An example of a concentric circles diagram, which is used to facilitate the mapping of a user’s current support networks (Stages 1 and 2). Figure S3. An example of suggestions of local resources that are presented to GENIE users, with location and further details (Stage 4) [file 40814_2020_553_MOESM2_ESM.docx]

**Additional file 2**

**
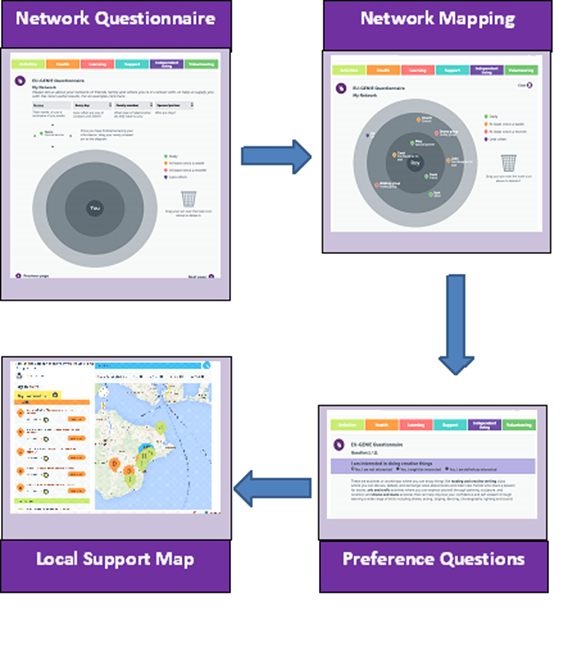
**

Figure 1. The stages of the GENIE intervention delivery.

**
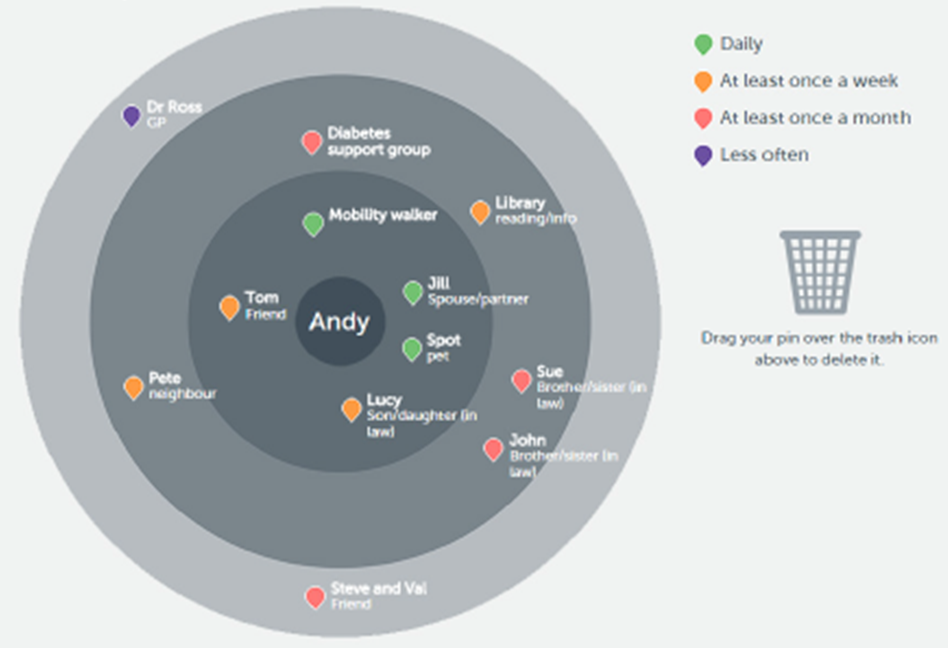
**

Figure 2. An example of a concentric circles diagram, which is used to facilitate the mapping of a user’s current support networks (Stages 1 and 2).

**
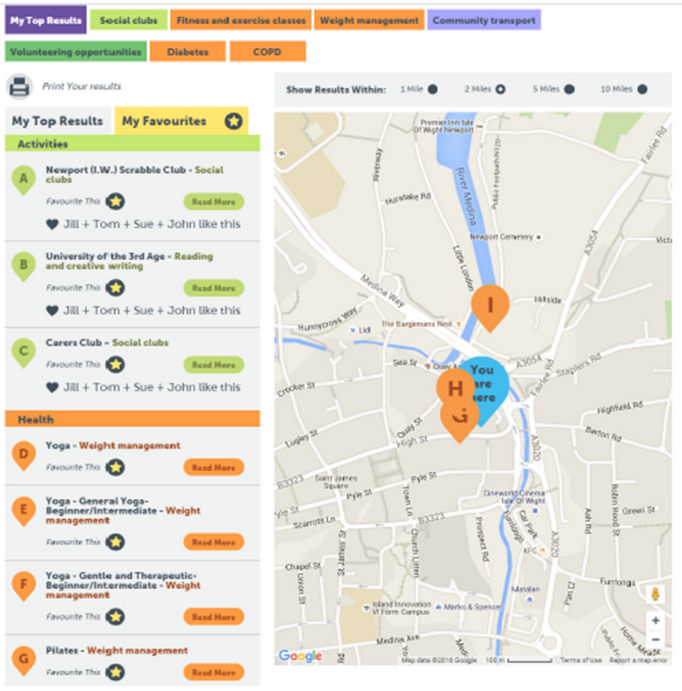
**

Figure 3. An example of suggestions of local resources that are presented to GENIE users, with location and further details (Stage 4).
