# Supplementary material for: Findings from an exploration of a social network intervention to promote diet quality and health behaviours in older adults with COPD: a feasibility study
Source: Pilot Feasibility Stud. 2020 Feb 6;6:15. doi: 10.1186/s40814-020-0553-z (PMC7003327; doi:10.1186/s40814-020-0553-z)
Supplement: Supplementary file 3 — Additional file 3. Video clips used for observational data [file 40814_2020_553_MOESM3_ESM.docx]

**Video clips used for observational data:**

<https://www.youtube.com/watch?v=qUgHb5kMxQ4>

<https://www.youtube.com/watch?v=f212f_14gcY>

<https://www.youtube.com/watch?v=DM-Q9UiBdVw>
